# Gold nanoparticles for cancer radiotherapy: a review

**DOI:** 10.1186/s12645-016-0021-x

**Published:** 2016-11-03

**Authors:** Kaspar Haume, Soraia Rosa, Sophie Grellet, Małgorzata A. Śmiałek, Karl T. Butterworth, Andrey V. Solov’yov, Kevin M. Prise, Jon Golding, Nigel J. Mason

**Affiliations:** 1 Department of Physical Sciences, The Open University, Walton Hall, Milton Keynes, MK7 6AA UK; 2 School of Medicine, Dentistry and Biomedical Sciences, Queen’s University Belfast, Lisburn Road, Belfast, BT9 7BL UK; 3 Department of Life, Health and Chemical Sciences, The Open University, Walton Hall, Milton Keynes, MK7 6AA UK; 4 Department of Control and Power Engineering, Faculty of Ocean Engineering and Ship Technology, Gdansk University of Technology, 80-233 Gdansk, Poland; 5 MBN Research Center, Altenhöferallee 3, 60438 Frankfurt, Germany

**Keywords:** Gold nanoparticles, Nanomedicine, Radiosensitisation

## Abstract

Radiotherapy is currently used in around 50% of cancer treatments and relies on the deposition of energy directly into tumour tissue. Although it is generally effective, some of the deposited energy can adversely affect healthy tissue outside the tumour volume, especially in the case of photon radiation (gamma and X-rays). Improved radiotherapy outcomes can be achieved by employing ion beams due to the characteristic energy deposition curve which culminates in a localised, high radiation dose (in form of a Bragg peak). In addition to ion radiotherapy, novel sensitisers, such as nanoparticles, have shown to locally increase the damaging effect of both photon and ion radiation, when both are applied to the tumour area. Amongst the available nanoparticle systems, gold nanoparticles have become particularly popular due to several advantages: biocompatibility, well-established methods for synthesis in a wide range of sizes, and the possibility of coating of their surface with a large number of different molecules to provide partial control of, for example, surface charge or interaction with serum proteins. This gives a full range of options for design parameter combinations, in which the optimal choice is not always clear, partially due to a lack of understanding of many processes that take place upon irradiation of such complicated systems. In this review, we summarise the mechanisms of action of radiation therapy with photons and ions in the presence and absence of nanoparticles, as well as the influence of some of the core and coating design parameters of nanoparticles on their radiosensitisation capabilities.

## Background

Cancer is one of the leading causes of death worldwide and the number of cancer-diagnosed patients is rapidly increasing, in part due to an ageing population, and is expected to reach 22 million cases in the next two decades (Stewart 2015). Currently, the main therapeutic approaches used to treat cancer are surgery, chemotherapy, and radiotherapy, delivered separately or in various combinations (Sánchez-Santos 2012).

Surgery and radiotherapy are key players for treating primary non-metastasised solid tumours, but for patients with co-morbidities that are unfit for surgery, deep-seated tumours, especially those associated with major blood vessels, or brain tumours, combined chemotherapy approaches are common.

In chemotherapy, pharmaceutical compounds that exert a cytotoxic effect disrupting mechanisms underpinning the rapid overgrowth of malignant cells are administered (Hanahan 2011; Joiner and van der Kogel 2009; Crawford 2013). Conventional chemotherapy is effective but also well-known for its severe side effects owing to the partially non-selective uptake of the chemotherapeutics both into healthy and cancerous cells in tissues and organs. Significant improvement has been made in recent years with the advent of nanomedicine, which provided an important addition to chemotherapy as a new medicine (Sun et al. 2014; Danhier et al. 2010).

Radiotherapy is a key treatment and is beneficial in the treatment of about 50% of all cancer patients (Delaney and Barton 2015). Such treatment relies on the deposition of energy (the dose) in tumour cells, typically by irradiation with either high-energy gamma rays or X-rays (photons), or energetic beams of ions, sufficient to damage the cancer cells or their vasculature and thus induce tumour death or nutrient starvation. However, like chemotherapy, photon radiotherapy is non-specific, since a significant dose can be delivered to healthy tissue along the track of the photons, in front and behind the tumour (Greish 2007; Hainfeld et al. 2008).

**Fig. 1:**
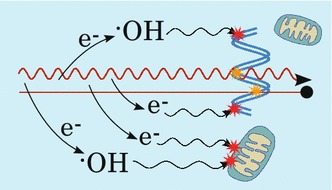
Illustration of mechanisms of radiation damage. Both photon and ion radiation (*red wiggly* and *straight lines*, respectively) may directly damage DNA (marked with *yellow stars*) or other parts of the cell, such as mitochondria (damage not shown), as well as ionise the medium thereby producing radicals and other reactive species (represented here by the ${\cdot }$OH radical) as well as secondary electrons, which can cause indirect damage after diffusion (*red stars*). Secondary electrons may also react with the medium to further increase the number of radicals. See text for further details

For radiotherapy, the central pathways to increase the therapeutic index, i.e. the ratio of treatment efficacy to side effects, are reversal of radiation resistance in tumour tissue, enhancement of radioresistance in healthy tissue, increasing radiosensitisation in tumour tissue, and better confinement of the deposited dose to the tumour volume (Kwatra et al. 2013). In this review, we will focus on the latter two pathways through the use of nanoparticles to achieve radiosensitisation and ion beam radiation to achieve a higher, more localised dose. The underpinning research involved in this area is highly multidisciplinary, including such diverse fields as atomic cluster physics, collision studies, materials research, nanoparticle synthesis, analytical chemistry focused on characterisation of the bio-nano interactions between the nanoparticles and the biological environment as well as mechanistic in vitro and in vivo studies. This is all aided by advanced imaging and by computational efforts to model the interactions between ions, biological matter, and nanoparticles.

In this review, we would like to sum up some key findings of the newly developed radiotherapy involving gold nanoparticles and bring up some of the mechanisms discovered and methodologies developed. This interdisciplinary research attracts a lot of attention from various communities, thus providing both experimental and computational insights into investigations from molecular to cellular level.

**Fig. 2:**
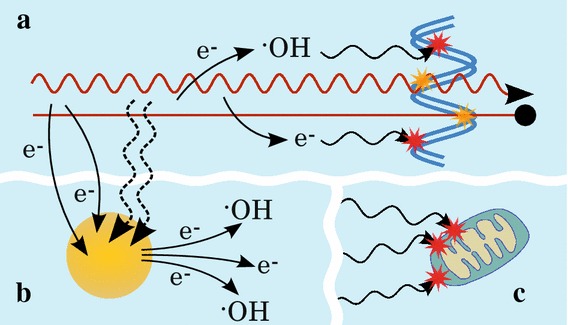
Illustration of mechanisms of radiation damage in the presence of nanoparticles. In addition to the direct and indirect damage (*yellow* and *red stars*, respectively) to DNA or other parts of the cell (**a**), the incident radiation may also interact with NPs (**b**) (illustrated by *dashed*, *wiggly arrows*) and induce the emission of secondary electrons which can then react with the medium to increase the production of radicals and other reactive species (like ${\cdot }$OH radicals); secondary electrons produced by the radiation or by NPs may also induce further electron emission from NPs. **c** All the secondary species may diffuse and damage other parts of the cell (like mitochondria). See text for further details

The review is organised as follows: First we present the principal mechanisms of radiotherapy using both photons and ions. Then the mechanisms of using nanoparticles (NPs) to achieve radiosensitisation is presented followed by the influence of several physico-chemical properties of such NPs (size, material, coating, charge) and their impact on toxicity and biodistribution. Finally, the paper will conclude with a brief summary of the field and future challenges.

## Conventional radiotherapy

### Principles of radiotherapy

Radiotherapy treatments rely on the deposition of energy along the path of the incident radiation. A series of events occur on different time scales after irradiation of biological medium and these can, in general, be referred to as the physical, chemical, and biological stages.

During the physical stage, the photons (or ions) interact with the medium, depositing energy, and either directly damage the cell by ionising fragmentation of the DNA or generate secondary species, such as low energy electrons or radicals, that can further damage DNA. This happens within the sub-femtosecond time scale.

**Fig. 3:**
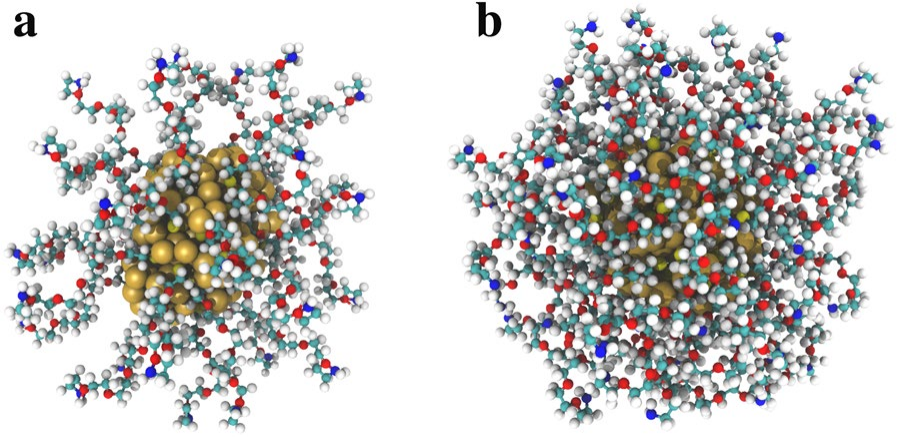
Illustration of PEG-coated AuNPs. Output from simulation of 1.4 nm AuNPs coated with **a** 32 and **b** 60 PEG molecules. Details in Ref. Haume et al. (2016)

Although damage to other parts of a cell than DNA can also lead to its death [for example, damage to mitochondria (Kobayashi et al. 2010; Pan et al. 2009)], the most widely used proxy for determining the efficacy of radiotherapy is to monitor the amount of DNA strand breaks produced. For in vitro work, using plasmid DNA is especially prevalent (Wyer et al. 2009; Folkard et al. 1993; Śmiałek et al. 2013, 2014).

Several studies have shown that the main pathway of DNA damage, from both X-rays and gamma rays, is through the production of water radicals with ca. 70% of damage caused by radicals and other reactive species—the most important being ${\cdot }$OH, NO${\cdot }$, H${\cdot }$, and $\mathrm{H}_2\mathrm{O}_2$—and 30% due to secondary electrons and direct fragmentation of the DNA (Kavanagh et al. 2013; Nikjoo et al. 2001; Emfietzoglou et al. 2005; Kwatra et al. 2013). These highly reactive radicals, particularly ${\cdot }$OH, can rupture molecular bonds and oxidise DNA or proteins of intracellular structures, such as mitochondria or membranes, which affects the stability of the cell membranes and organelles. In addition to the damage produced by radicals, it has been shown that secondary electrons, produced in ionisation events, can cause DNA strand breaks as well through a process known as dissociative electron attachment (Pan et al. 2003). Secondary electrons can also further increase the radical yield by interacting with the water medium as shown in Fig. 1. The damage due to secondary species is referred to as the indirect damage and takes place at the chemical stage (times within microseconds after irradiation). And finally, during the biological stage (from microseconds and up), the damaged cells exhibit the consequences of the radiation exposure. If exposed to a sufficiently high radiation dose, with appropriate dose rate and dose fractionation, the cell will die.

The damage caused to DNA by radiation can result in a range of various lesions, including base damage, single strand breaks (SSBs) or, less frequently, double strand breaks (DSBs). In most cases, base damage and SSBs can be effectively repaired by the cell repair mechanisms, whereas DSBs, especially when induced at high levels (often referred to as “clustered” or “complex” damage), are difficult to get successfully repaired and are therefore more damaging to cells (Kobayashi et al. 2010).

The DNA may be successfully repaired ensuring continued, normal cell proliferation and function, or may result in misrepair, which in turn can lead to either its death or to mutations with a possibility of transforming a normal cell into a cancerous cell. Thus increasing the amount of clustered damage as well as sparing healthy tissue is the motivation for seeking a better optimisation of radiation damage during treatment.

Herein, we focus on the external photon and ion beam radiotherapy and their interaction with nanoparticles. The main difference between these two types of radiation lies in their ways of interaction with matter and modalities of the formation of the secondary species.

### Photon-based radiotherapy

In cancer radiotherapy, photons generally refer to either gamma or X-rays, with X-ray energies usually in the range between 80 keV and 25 MeV, predominantly in the 8–18 MeV range for modern high-energy linear accelerators that are used to produce and form the radiation beam (Dendy and Heaton 2011). The photons effectively ionise biological matter and due to the relatively simple setup required to produce them, proton-based treatment protocols have been used with great success for decades.

A sufficient dose can kill any tumour cell but the concerns of the amount of damage to healthy tissue in the vicinity of the tumour limit how much radiation a patient can receive (Porcel et al. 2014; Hainfeld et al. 2008). Gamma and X-rays have an exponential dose deposition with tissue depth, therefore a significant fraction of the total dose is delivered to healthy tissue in front of and behind the target (Hainfeld et al. 2008; Krämer and Durante 2010). In an attempt to increase the dose delivered to the target while keeping the dose delivered to surrounding, healthy tissue tolerable, lower-energy radiation from multiple angles can be used. The overlap of the beams on the cancer tissue ensures a high total dose on the target, but invariably leads to a much larger volume of low-dose-irradiated healthy tissue (Kobayashi et al. 2010).

An important disadvantage of photon therapy is that cancer tissues can have, or develop, resistance to radiation; moreover, due to the physical extent of this tissue, it can be difficult to effectively irradiate the whole tumour, which may lead to its regeneration (Kwatra et al. 2013).

### Ion-based radiotherapy

To overcome the abovementioned disadvantages associated with the photon-based radiotherapy, the therapy based on ions as an alternative method achieved the lowering of the unwanted effects on healthy tissues and better control of the size and shape of the irradiated volume.

Ion radiotherapy refers to the use of ion beams as the radiation source, consisting of ions of hydrogen (protons), helium, carbon, or oxygen. Usually, hydrogen and helium ions are referred to as “light ions” and the others as “heavy ions”.

The attractiveness of ion radiation stems from the characteristic interaction properties with matter, namely that the energy deposition is maximum for low ion velocities. This means that the energy deposition of an ion increases as it traverses tissue, producing radicals and secondary electrons along the track. This causes a strong enhancement of the linear energy transfer (LET, energy deposited per unit distance traversed in units of keV/$\upmu$m) near the end of the track in what is called the Bragg peak (Tsujii et al. 2014). By tuning the incident energy of the ion, the location of the Bragg peak, which extends over only a couple of millimetres, can be directed into the tumour tissue leading to a very high, well-defined, and localised dose. For this reason ion-based therapy is considered superior when tumours are situated close to sensitive tissues or vital organs such as the spinal cord, brain, and eye (Kraft 1990). During therapy, a range of ion energies are delivered, leading to the production of a Spread-Out-Bragg-Peak (SOBP) (Krämer et al. 2000). This increases the volume that can be treated but it also leads to a higher amount of radiation received in the entrance channel (the healthy tissue preceding the target).

The amount of deposited energy depends on the mass and energy of the incident ion, with heavier ions having, in general, a higher LET (Scifoni et al. 2010). However, irradiation with heavier ions tends to deposit some energy beyond the target due to fragmentation of the incident ion near the Bragg Peak. This may produce lighter nuclear fragments which continue depositing some energy, potentially causing further damage to healthy tissue (Haettner et al. 2006).

The prediction of cell killing is not straightforward. When the LET of incident ions increases, the radical production increases as well, which may lead to a reduced number of radicals available to interact with the cell due to recombination of these radicals with each other (Usami et al. 2010; Terato et al. 2008).

### Modelling radiation response

To make predictions of radiation effects, a popular simulation model of the effect of radiotherapy, local effect model (LEM) is used (Elsässer and Scholz 2007; Elsässer et al. 2008). The premise of the LEM is that the local biological response to radiation is equal for equal doses and independent of the type of radiation. If the biological effect of a given dose is known for photon irradiation, then the biological effect of ion radiation can be calculated once the dose is known. The dose can be calculated from the LET of a given ion, which is known from experiments, and can be implemented to the model. The LEM model has shown good agreement with experiments (Krämer and Durante 2010; Elsässer et al. 2008; Combs et al. 2009).

Surdutovich and Solov’yov took another approach and formulated the so-called multiscale approach to the physics of radiation damage with ions (Surdutovich and Solov’yov 2014). In this framework, all physical interactions (e.g. ionisation of matter as well as the production and transport of reactive species such as secondary electrons and free radicals) between ions and matter are accounted for quantitatively, which opens up a possibility to evaluate and predict biological consequences of radiation damage. The multiscale approach combines the information on the production of secondary species, their energy spectra, the transport of secondary species, and the interaction cross sections between electrons and radicals and biological matter. By thorough understanding of these nanoscale processes, one is able to evaluate the probability of single and double DNA strand breaks as well as complex DNA lesions to be formed upon irradiation. From these data, survival curves can be obtained as a function of deposited radiation dose (Surdutovich and Solov’yov 2014). In this way, the multiscale approach bridges the femtosecond and sub-nanometre scale up to the biological timescale and dimensions. In a recent publication, the multiscale approach has been proved to successfully predict the survival probability of a number of mammalian cancerous and normal cell lines and some related phenomena, for example oxygen enhancement ratio (Verkhovtsev et al. 2016).

One of the important phenomena predicted and elaborated within the multiscale approach is the thermomechanical pathway of biodamage. It is caused by intense heating of the medium due to relaxation of the energy deposited by projectile ions to the molecules of the medium (Surdutovich and Solov’yov 2010; Surdutovich et al. 2013; de Vera et al. 2016). A rapid increase of temperature and pressure close to the ion’s path causes the formation of a shockwave which may damage DNA or other parts of the cell directly and increase the transport of reactive chemical species. Thermomechanical effects were recently shown to be important in the prediction of ion-induced radiation damage thus they need to be accounted for (Verkhovtsev et al. 2016).

## Radiotherapy with gold nanoparticles

In addition to the use of NPs in nanomedicine, which has successfully improved the specificity of modern chemotherapeutics in recent years (Ranganathan et al. 2012; Danhier et al. 2010; Sun et al. 2014), promising demonstrations of the radiosensitising potential of NPs in the last decade, both in vitro and in vivo, now mean that significant research efforts focus on NPs for improved dose localisation for radiotherapy (Hainfeld et al. 2008; Kwatra et al. 2013; Porcel et al. 2012; Liu et al. 2010; Polf 2011).

Gold NPs (AuNPs) in particular, have become popular since they have several advantages, including good biocompatibility, straightforward synthesis in a wide range of sizes, and easy surface functionalisation by the attachment of ligands required to target cancer cells, and organelles therein, or improved life time in the bloodstream (McMahon et al. 2011; Hainfeld et al. 2008; Kwatra et al. 2013; Malam et al. 2009; Barreto et al. 2011; Carter et al. 2007; Liu et al. 2010). Additionally, as discussed later, AuNPs have a large interaction cross section with X-ray radiation up to about 1MeV as well as with ion radiation.

The contribution of AuNPs in increasing the radiotherapy efficiency is measured by the dose enhancement factor (DEF). The DEF of AuNPs is defined as the ratio of the radiation dose absorbed by the tumour cells in the presence of AuNPs to the dose absorbed in the absence of AuNPs (Muddineti et al. 2015). This may vary with the concentration and characteristics of the AuNPs and their location inside the cell (Butterworth et al. 2012; Hossain 2012).

Among other groups studying the benefit of AuNPs in combination with X-ray radiotherapy, Zhang et al. employed Monte Carlo simulations to show a possible radiosensitisation with AuNPs and found that radiation beam will deposit a lower dose after having passed through the AuNP containing region, thus increasing the therapeutic ratio (Zhang et al. 2009).

As previously mentioned, the main contribution to the killing of cells from radiotherapy is through the production of free radicals. This is also the source of radiosensitisation when using NPs as they increase the radical production (Porcel et al. 2010; Verkhovtsev et al. 2015a).

The interaction between NPs and radiation is divergent for photons and ions, and will be briefly reviewed below.

### Nanoparticles with photon radiation

X-rays and gamma rays interact with NPs mainly through the excitation and scattering of electrons of the NP (Hainfeld et al. 2008; Kobayashi et al. 2010). When the excited electron comes from an inner shell, the so-called Auger de-excitation processes are especially likely. This leads to one or more Auger electrons being emitted, the latter known as Auger cascades where more than 10 electrons can be emitted (Sancey et al. 2014; Porcel et al. 2010).

Auger electrons have energies below 5 keV and have been shown to be effective in damaging DNA directly in addition to ionising surrounding water molecules (Pan et al. 2003; Butterworth et al. 2013). All of the secondary electrons from the NPs may also interact with other NPs, resulting in further Auger electron emission (Kobayashi et al. 2010; Porcel et al. 2010) or they may be absorbed by the medium causing ionisation and radical formation (Hainfeld et al. 2008), see illustration in Fig. 2. Additionally, Porcel et al. suggested that the positively charged NPs (after emission of photo- or Auger electrons) could cause surrounding water molecules to become unstable and more easily dissociate, further increasing the radical yield in the environment of the NPs (Porcel et al. 2010).

One of the proposed reasons for the observed radiosensitisation of NPs under photon irradiation is their higher interaction cross section with the radiation up until the megavoltage range compared to that of the water and soft tissue of the cells which contributes to the localisation of the dose. Due to the photoelectric effect scaling proportionally to $(Z/E)^3$, where *Z* is the atomic number and *E* is the energy of the incoming photon, Auger emission is especially likely to take place for high-Z metals like gold, gadolinium, platinum, or silver (Kobayashi et al. 2010; Porcel et al. 2010; Coulter et al. 2013; Schlathölter et al. 2016), which have been shown to produce a larger number of Auger electrons when compared to the relatively light elements of biological tissue such as hydrogen, carbon, and oxygen. The increase in the interaction cross section of gold vs. soft tissue decreases at high energies, and it has indeed been found that the energy of the radiation plays a major role in the radiosensitisation effect. Rahman et al. found that low energy X-rays of 80 kVp (peak kilovoltage), in combination with AuNPs were able to deliver a high DEF and that the effect increased with increasing concentration of AuNPs. DEF values of 4, 20, and 24.6 were found for concentrations of 0.25, 0.5, and 1 mM, respectively. Furthermore, at 150 kVp X-ray, the DEF increased from 1.4 to 2.2 for 0.5 and 1 mM AuNPs, respectively (Rahman et al. 2009).

In the clinical context, radiotherapy is often delivered using MeV X-rays, since keV photons have less penetration in tissue and would only be able to treat superficial tumours (Rahman et al. 2009). Despite the much lower interaction cross section at these energies, experiments show a radiosensitisation effect nonetheless (Butterworth et al. 2013). McMahon et al. (2011) showed computationally that the radiosensitisation seen with photons in these energies is caused by the interaction of NPs with secondary species produced by ionisation of the water medium rather than with the radiation itself.

Particularly efficient for cell killing is the induction of “complex damage” due to the difficulty in successfully repairing such damage (as described above). For example, Porcel et al. showed an increase in the DSB/SSB ratio in plasmid DNA when using platinum NPs with ion radiation (Porcel et al. 2010), and increased amount of strand breaks was also observed by Xiao et al. (2011) who used AuNPs irradiated by electrons.

The local effect model (LEM) was used by Lin et al. (2015) to simulate the effect of AuNPs under X-ray and proton radiation on cell killing. Their study showed that the uptake of NPs into cells is crucial for proton therapy but less so for photon therapy where AuNPs located in the intercellular medium can generate radicals that migrate and contribute to DNA damage. It should be mentioned that the simulation only included DNA damage, which may not be the only target in the cell and furthermore, it accounted only for direct interaction between the radiation and the NPs, thus excluding the interaction between NPs and secondary species, as described above.

However, it should be noted that although producing DNA strand breaks is an important factor in inducing cell death and most experiments monitor the increase in SSBs and DSBs, it is by no means the only target in cancer cells for nanoparticle radiosensitisation (Kobayashi et al. 2010; Štefancikova 2014; McQuaid et al. 2016).

Another potential target is the mitochondria, as disruption of their membrane potential can lead to apoptosis. AuNPs have been found to induce oxidation of the mitochondrial membrane protein cardiolipin and also the disruption of mitochondrial membrane potential. Depolarisation of the membrane potential may be due to increased radical production promoted by NPs themselves, and oxidation of cardiolipin causes the release cytochrome c. Both processes can trigger apoptosis and thus contribute to enhanced radiosensitisation (Taggart et al. 2014, 2016).

### Nanoparticles with ion radiation

In addition to the interaction between NPs and the secondary electrons produced by the ionisation of the medium by the ion radiation, it was recently shown in a theoretical study that metal NPs in combination with ion radiation significantly increase the secondary electron yield, compared to that of pure water, due to excitation of plasmons in the NP Verkhovtsev et al. (2015a, b).

Plasmons are the excitations of delocalised electrons of the material and can be efficiently excited especially in metals. Verkhovtsev et al. showed that an order of magnitude increased the production of low energy electrons from metal NPs, compared to a similar volume of water, as the result of collective electronic excitation in the NP Verkhovtsev et al. (2015a, b). Specifically, it was shown that noble metal NPs are superior to, for example, gadolinium NPs because of the energy of the surface plasmon, which in noble metals is higher than the ionisation potential, such that the relaxation of plasmon excitations can cause the emission of an electron.

The effect of collective excitation was also shown for carbon-based NPs, although the effect is not as strong as for other metal systems like gold or platinum (Verkhovtsev et al. 2015c). Due to the fact that the plasmon resonance energy for carbon NPs occurs at higher energies than for NPs of noble metals, it was proposed that NPs made of a combination of materials with different plasmon resonance energies will be able to exploit a larger spectrum of ion energies, leading to a more efficient electron production from such NPs.

## Physico-chemical properties of NPs and their role in radiosensitisation

 There are several aspects to consider when developing new NPs such as the material they should be constructed from, their shape and size, the surface coating, and the net charge on the NP.^1^ All of these parameters influence cellular uptake and the biological response of cells as well as their interaction with radiation. Finding the optimal design is a non-trivial problem due to the large number of tuneable parameters. Here, we will overview some of the key parameters.

### Size

The size of NPs used for radiosensitisation affects both how they interact with the biological system and how they interact with the radiation.

The biodistribution and route of elimination from the body are strongly depending on the size of the NPs. To avoid accumulation of NPs in organs such as heart and liver, causing potential long-term side effects, metal NPs should be eliminated from the body within a few days, which will still provide a window for radiotherapy with NPs present. This is best achieved through renal clearance which is dependent on the size of the NPs (Alric et al. 2013; Barreto et al. 2011; Sancey et al. 2014). NPs with a hydrodynamic diameter greater than 10 nm are more likely to be captured by the liver, whereas NPs smaller than 6 nm are usually eliminated by renal clearance independently of their charge (Longmire et al. 2008; Bertrand and Leroux 2012; Moghimi et al. 2012; Alexis et al. 2008; Almeida et al. 2011; Albanese et al. 2012; Owens and Peppas 2006; Choi et al. 2007). NPs between 6 and 10 nm can also be eliminated via renal clearance, although in this case, positively charged NPs are eliminated faster than negative or neutral NPs (Longmire et al. 2008).

Although current data point to a maximum cell uptake by NPs between 20 and 60 nm (Albanese et al. 2012; Chithrani et al. 2006; Zhang et al. 2009; Perrault et al. 2009), smaller NPs still accumulate in tumours due to the enhanced permeability and retention effect (EPR) (Sancey et al. 2014). Smaller NPs will also tend to diffuse further into tumour tissue from the bloodstream, and therefore present a more even distribution in larger tumours than larger NPs. This may counteract the lower active uptake and the faster elimination from the blood stream of small NPs (Albanese et al. 2012; Perrault et al. 2009).

Regarding toxicity of AuNPs, some studies have concluded that toxicity is minimal for NPs below 5 nm and above 50 nm, but severe at intermediate sizes (Akhter et al. 2012). Other studies have shown toxicity for AuNPs of diameter 3, 8, and 30 nm, but not for 5, 6, 10, 17, or 48 nm (Vijayakumar and Ganesan 2013). This is clearly a complex question, and further research is necessary to define mechanisms of toxicity of AuNPs.

When considering the interaction between NPs and radiation, the size of the NPs is also important. As AuNPs become larger, more of the ionising events from interaction with secondary electrons and radiation occur in the bulk of the NPs, reducing the dose deposited in the medium around the NP (McMahon et al. 2011). Carter et al. (2007) found that the production of low energy electrons was increased for 3 nm NPs compared with 6 nm NPs, and Lin et al. (2015) found improved cell killing in their theoretical study for 2 nm AuNPs compared to sizes up to 50 nm because secondary electrons formed in larger NPs have a higher probability of dissipating their energy inside the NP before reaching the surface.

### Surface charge

A positive charge on the surface of NPs is thought to improve the uptake into cells due to its interaction with the negatively charged lipid membrane (Beddoes et al. 2015; Albanese et al. 2012; Yah 2013; Hirsch et al. 2013; Kalay et al. 2014). Positively charged NPs could also selectively target cancer cells because of the glycocalyx structure, which, besides often being larger, can be more negatively charged on some cancer cells (Stylianopoulos et al. 2013; Sarin 2010). This glycocalyx is composed of different glycoproteins, and glycosaminoglycans, which can influence the membrane organisation, signal transduction, and possibly enhance endocytosis (Paszek et al. 2014).

Although the amount of charge on NPs is linked to the cell membrane penetration, the exact optimal amount of charge is unknown (Beddoes et al. 2015). Da Rocha et al. (2013) showed computationally that the uptake pathway is dependent on the amount of charge and for neutral or slightly cationic NPs, a passive membrane translocation was favoured, whereas for highly cationic NPs, an endocytosis-mediated uptake was dominant. Due to the more pronounced interaction, positively charged NPs induce higher local distortion of the membrane and can perturb the transmembrane potential thereby interfering with certain cell functions, such as ion transport, and increase the probability of pore formation in the membrane (Beddoes et al. 2015; Albanese et al. 2012).

When a foreign object is introduced to the bloodstream, specialised serum proteins called opsonins will adsorb onto the surface of the object, labelling it for clearance from the body (Malam et al. 2009). Since these proteins have a negative charge, positively charged NPs will tend to be eliminated faster in vivo compared to neutral or negatively charged NPs (Alric et al. 2013; Alexis et al. 2008). This can be circumvented by appropriately coating the NP, as will be discussed below.

### Concentration of NPs

It was recognised by Hainfield et al. (2004) in some of the earliest studies of NPs as a radiotherapy agent, that the concentration of NPs in tumour tissue plays an important role in the radiosensitisation effects, and it has since been reported that the concentration of AuNPs plays a larger role in radiation dose enhancement than their size (Mesbahi et al. 2013; Babaei and Ganjalikhani 2014). Brun et al. investigated the relationship between plasmid DNA:AuNP ratio, incident X-ray energy, and AuNP size (Brun et al. 2009). Across a range of DNA:AuNP ratios between 1:1 and 1:10, photon energies from 14.8 to 70 keV, and sizes in the range of 8–92 nm, they found that the best radio-enhancement (sixfold improvement relative to the controls) was achieved with 37.5 nm AuNPs, at ratio of 1:1 DNA:AuNP, and an energy of 50 keV (Brun et al. 2009).

## Coating of nanoparticles

Coating of NPs can to help control the interaction of NPs with the proteins of the bloodstream (Monopoli et al. 2011, 2012; Krpetić et al. 2014). Additionally, NP coating can be used to target specifically the tumour cells in the body (see “Active targeting” section and references therein). The targeting strategies that are being employed in order to ensure a sufficient concentration of NPs in tumour cells can be divided into two categories: passive targeting and active targeting (Akhter et al. 2012). In passive targeting, one takes advantage of the higher endocytic uptake of cancer cells and leaky vasculature around tumours which allow for higher uptake of NPs than in healthy tissues (Barreto et al. 2011), while in active targeting, the NPs are functionalised with specific molecules that interact with receptors known to be selectively present in tumour cells (Salvati et al. 2013).

The applied coating also allows for controlling of the charge of the surface of the NPs. In addition to the interactions with opsonin proteins, that were already mentioned, the surface charge plays a role in the stability of AuNPs (for example, their tendency to aggregate) in aqueous solution and in the body (Alkilany and Murphy 2010). Coating of NPs can therefore provide partial control of life time and uptake dynamics of the AuNPs (Chithrani et al. 2009; Thierry and Griesser 2012; Saptarshi et al. 2013; Krpetić et al. 2011). One concern, however, when applying coating to NPs intended for radiosensitising agents is that the coating may absorb secondary electrons emitted from the metal core. Although radiosensitisation has been shown for coated AuNPs (Liu et al. 2010; Zhang et al. 2012), the coating may reduce the amount of radicals produced in the process, as recently shown by Gilles et al. (2014).

### Passive targeting

When the organism recognises a foreign body in the bloodstream, specialised serum proteins called opsonins will adsorb on the surface of the body labelling it for clearance from the body (Malam et al. 2009). It has been shown that this can be prevented by attaching appropriate molecules on the surface of the NPs, for example poly(ethylene glycol) (PEG) (Alexis et al. 2008; Otsuka et al. 2003; Illés et al. 2014) (see Fig. 3 for an illustration of a PEG-coated AuNP). It is thought that PEG-coating of NPs provides a repelling force on the opsonins thus unlabelling them to cover their surface (Thierry and Griesser 2012; Otsuka et al. 2003). Since NPs tend to concentrate in tumour tissue as a consequence of abnormal blood vessel wall formation around tumour tissue and poorly developed lymphatic system that limits drainage of macromolecules from tumour tissue (Ranganathan et al. 2012), the enhanced permeability and retention effect (EPR) is observed in this case. Increasing the blood circulation time by coating with e.g. PEG thus leads to higher passive uptake due to the EPR effect. The ability of the coating layer to provide the passive targeting conditions depends on several factors, like the size of the NP core or the length and surface density of capping molecules and have been already investigated both computationally and experimentally (Otsuka et al. 2003; Walkey et al. 2012; Kingshott et al. 2002; Haume et al. 2016; Lee et al. 2009).

### Active targeting

Active targeting involves attaching to the surface of NPs other molecules that have specific affinities to interact with cancer tissues. The main motivation is to avoid relying on passive uptake through the EPR effect (Coulter et al. 2013). This has been achieved, for example, with antibodies (Shmeeda et al. 2009), peptides (Chanda et al. 2010; Kumar et al. 2012), folates (Samadian et al. 2016; Zwicke et al. 2012), aptamers (Li et al. 2015; Wu et al. 2015), hormones (Dreaden et al. 2009, 2012), and glucose molecules (Calvaresi and Hergenrother 2013; Gromnicova et al. 2013; Hu et al. 2015).

### Combination targeting

It is possible to combine the two abovementioned targeting strategies. To utilise PEG for increased circulation time, the ratio of PEG to targeting ligand has to be optimised. An excess of targeting ligand will lead to reduced circulation time (Shmeeda et al. 2009), whereas an excess of PEG will dilute the effect of the active targeting groups. Dai et al. found that for combination coatings, the length of PEG molecules should not exceed the length of the targeting ligands in order to prevent PEG molecules blocking the receptor–ligand interaction (Dai et al. 2014).

## Gold nanoparticle toxicity

Despite the various advantages of AuNPs, they are relatively expensive and even if AuNPs are reported to be inert and biocompatible, more information about their toxicological profile still needs to be provided (Kwatra et al. 2013). As mentioned above, NPs below 5 nm are often used for radiosensitisation purposes due to the relatively rapid elimination from the body, good uptake, and favourable interaction with radiation, but at these sizes AuNPs can become chemically reactive (Alkilany and Murphy 2010; Ionita et al. 2007; Zhang et al. 2003; Pan et al. 2009; Xia et al. 2006).

Previous work has focused on the potential toxicity of AuNPs. Tables 1 and 2 form a non-exhaustive list of the different toxicology studies conducted with differently sized AuNPs, various cell types, and using ranges of AuNPs concentrations. This toxicity can be measured on in vitro models, using different measurements. The most common one is the measure of the half maximal inhibitory concentration ($\mathrm {IC_{50}}$), the concentration of chemicals which gives a decrease of 50% of the cell viability.

**Table 1:** In vitro toxicology studies of cancer models to AuNP exposure for AuNPs smaller than 4 nm

Size (nm)	Coating	Cancer cell line	Exposure	Time (h)	Toxicity	Ref.
2	MMPC1	COS-1	0.38–3 μM	1–24	IC_50_ = 1.0 μM	Goodman et al. (2004)
	MMPC2				IC_50_ > 7.37 μM	
	MMPC1	Red blood cells	0.27–833 μM		IC_50_ = 1.1 μM	
	MMPC2				IC_50_ = 72 μM	
3.5 ± 0.7	Lysine, poly(lysine)	RAW 264.7 mouse macrophage	10–100 μM	24–72	>100 μM after 24 h	Shukla et al. (2005)
1.4	PH_2_PC_6_H_4_SO_3_H	MV3,	<0.4 mM	24	IC_50_ = 0.24 μM	Tsoli et al. (2005)
		BLM			IC_50_ = 0.30 μM	
1.1	GSH	HeLa	5.6 mM	48	IC_50_ = 3130 μM	Pan et al. (2009)
1.4	TPPMS				IC_50_ = 48 μM	
	TPPMS, GSH				IC_50_ = 181 μM	
1.4	TPPMS	HeLa	Up to 10 mM	36	IC_50_ = 30 μM	Pan et al. (2007)
		SK-mel-28			J774A1 IC_50_ = 30 μM	
					L929 IC_50_ = 56 μM	
	TPPTS	HeLa			IC_50_ = 46 μM	
1.9		BAECs	0.125-1 mM		~30% cell death at 1 mM AuNPs exposure	Rahman et al. (2009)
1.9		Du-145	Up to 2 mg/ml	24	LD_50_ = 20 μM	Coulter et al. (2012)
		MDA-MB-231			LD_50_ = 24.6 μM	
		L132			LD_50_ = 320 μM	

*MMPC1 and MMPC2* mixed monolayer gold clusters functionalised with quarternary ammonium and with carboxylic acid, respectively,* TPPMS* sodium triphenylphosphine monosulfonate,* TPPTS* sodium triphenylphosphine trisulfonate,* PEG* polyethylene glycol,* GHS* glutathione

**Table 2:** In vitro toxicology studies of cancer models to AuNP exposure for AuNPs larger than 4 nm

Size (nm)	Coating	Cancer cell line		Exposure	Time (h)	Toxicity	Ref.
4, 12, 18	CTAB, citrate, cysteine, glucose, biotin	K562 leukaemia	Human	1–250 nM	72	>25 μM	Connor et al. (2005)
4.8	PEG	HeLa		1–250 μM	48	IC_50_ = 0.205 mM	Zhang et al. (2012)
12.1						IC_50_ = 0.477 mM	
27.3						IC_50_ = 0.448 mM	
46.4						IC_50_ = 0.613 mM	
33	CTAB, citrate	A549		0–120 nM	48	IC_50_ $\approx$ 100 nM	Patra et al. (2007)
		BHK21				No toxicity observed for BHK21 up to these concentrations	
35.6 ± 6.7	Cetuximab antibody	Panc-1		100 nM		IC_50_ $\approx$ 100 nM	Glazer et al. (2010)
		Cama-1				NA	

*CTAB* cetyltrimethylammonium bromide,* PEG* polyethylene glycol

From the tables, it can be concluded that the toxic potential of AuNPs varies depending on their size and applied coating. Moreover, the toxic potential can also differ for various cell types. Pan et al. (2007) showed that the toxicity of coated AuNPs is size-dependent but does not depend on the type of coating as, for example, sodium triphenylphosphine monosulfonate (TPPMS) and sodium triphenylphosphine trisulfonate (TPPTS) coatings have the same toxicity for different cell lines.

However, Tsoli et al. (2005) found 50% toxicity after exposure to 0.24 μM of 1.4 nm AuNPs for 24 h. They found that AuNPs can improve the toxicity for cancer cell as compared to standard chemotherapy. For example, the $\mathrm {IC_{50}}$ on a melanoma cell line exposed to 1.4 nm AuNPs was 180 times lower than the $\mathrm {IC_{50}}$ after exposure to cisplatin (Tsoli et al. 2005).

Furthermore, altered gene expression has also been observed due to the presence of NPs causing phenotypic changes (Ng et al. 2015) and cytokine induction (Fujiwara et al. 2015). NPs may also have a role in propagating the bystander effect (Thubagere and Reinhard 2010). The bystander mechanism is observed when non-irradiated cells behave as if they were irradiated due to signals received from their irradiated neighbours. This effect is mainly propagated through reactive nitrogen and oxygen species, oxidised DNA from apoptotic cells, and cytokine production and release (Havaki et al. 2015). Since NPs seem to interfere with these mechanisms it is possible that they could potentially propagate bystander signalling. Thus determining the way NPs interact and modulate cell response could give further insights towards targeting specifically cancer cells and improving therapeutic outcomes.

## Future challenges and outlook

Even though photon radiotherapy is the most common treatment for a number of cancers with high effectiveness, it can still be optimised in order to reduce the side effects and increase the survival of healthy tissue.

It has already been shown that introducing various radiosensitisers may help to achieve this goal, and among others, NPs present a high potential for various modes of action in the cancerous cells. Nonetheless, although increasing radiotherapy efficacy using nanoparticles could potentially improve this survival rate in the clinic context, in this still-developing field there are many unknowns in the mechanisms of action both at the molecular and cellular level, as well as when considering their potential impact on cellular communication.

Increased radical production has been attributed to the presence of NPs even in the absence of radiation which can cause damage to the cellular components due to ROS being generated (Pan et al. 2009) triggering first the apoptosis and as a consequence the necrosis of the cell (Xia et al. 2006). Although numerous studies on the size, shape, and capping agent of NPs have been performed, it is still not clear what are the optimal conditions for the highest targeting rate of cancerous cells; thus much more work in this field is required.

From the very basic knowledge of photoelectric and related effects it can be easily concluded, that there are clearly benefits in combining AuNPs with radiotherapy. Here likewise much work is still necessary in order to optimise not only the multi-parameter properties mentioned above, but also to predict the most efficient way in secondaries production. It was already shown that the surface modifications, which increase the cellular uptake and make the passive or active targeting possible, may cap the secondary electrons in the close vicinity of the NP, thus preventing an efficient radiosensitisation. This implies that some new compromises between what has been known to work and the aimed actions must be explored.

There is a great amount of both experimental and theoretical work devoted to all possible parameters of NPs. Such great variability of sizes, shapes, and coatings associated with the differential cellular responses dependent on cancer types makes it at the moment difficult to establish any correlations or standard conditions for treatments; therefore, some clarification and organisation of the achievements of various communities must be done.

## Abbreviations

AuNP: gold nanoparticle
CTAB: cetyltrimethylammonium bromide
DEF: dose enhancement factor
DSB: double strand break
EPR: enhanced permeability and retention
GHS: glutathione
IC_50_: half maximal inhibitory concentration
LEM: local effect model
LET: linear energy transfer
MMPC1: mixed monolayer gold clusters functionalised with quarternary ammonium
MMPC2: mixed monolayer gold clusters functionalised with carboxylic acid
NP: nanoparticle
PEG: polyethylene glycol
SOBP: spread-out-Bragg-peak
SSB: single strand break
TPPMS: sodium triphenylphosphine monosulfonate
TPPTS: sodium triphenylphosphine trisulfonate

## Footnotes

1 Changed “Parameters of” to “Physico-chemical properties of” since this was requested by the reviewer for the paragraph in the introduction, and so to keep it the same.
